# Popular Appreciation of Dentistry

**Published:** 1886-10

**Authors:** L. C. Ingersoll


### POPULAR APPRECIATION OF DENTISTRY.

BY L. C. INGERSOLL, D. D. S.

READ BEFORE THE ODONTOLOGICAL SOCIETY OF WESTERN PENNSYLVANIA.

Every current of thought running through the popular mind has, at its source, originating causes. We can best learn how to deal with the popular sentiment concerning dentistry by inquiring into its origin and development. Ideas have a development from a germ, the same as do organic and tangible things. Let me start out, therefore, with a few germinal thoughts.

1. It is impossible to dissociate a man and his calling. In some countries of Europe and the old world, the calling makes the man. The social life, the business relations and the accumulated results of a man's life, are determined by his calling. In the early days of this country, the same was true here. Certain callings were honorable and highly esteemed, and the work of men engaged in them was highly appreciated. But it was well understood that men desiring to enter these high and honorable callings must be specially qualified for them. When once initiated, the calling itself made the esteem and wealth of the community accessible to the man holding the position.

In these modern days of democratic America, signs are changed; the man is not known by his calling. The calling does not make the man. Once, to call a man *Doctor*, was to fix the eyes of many passers-by upon him as a man to be highly esteemed for his work's sake. To call a man *Honorable*, was at once to place him high in the ranks of society. To call a man *Professor* was, in the popular mind, a perpetual endowment of learning. But now, when any one who deals in patent medicines, or sells corn plasters, calls himself, and is called by others, *Doctor*—and when a member of the common council of a small village has the title of *Honorable*, as well as the Senator of the United States—and when the barber and the writing master, as well as the learned collegiate, are called *Professor*, the popular sentiment is, that the man makes the calling—that the calling is just what the man makes it to be. The various pursuits in life are esteemed and appreciated for just what those who are engaged in them make them. *First* the man, *then* his calling.

2. That which is most highly appreciated among men is mental endowments and culture. It is not so much what a man does, as the amount of brains he takes into his work. Two men are engaged in railroading; one is the Irish plodder with his wheelbarrow, the other the genius who superintends and manages the road. The appreciation of the services of the one is measured by the $1.50 per day which he receives. The appreciation of the services of the other is measured by the $25.00 per day which he receives. The difference is created by the different mental capacities of the two men. It is not strength of muscle, cunning workmanship, deft fingers and manipulative skill, but the brain that employs the strength and guides the fingers, which can command a large remuneration for services. Examples like the foregoing are numerous.

3. Dentists are no exception to the general rule. The man who puts the most brains into his work can command the highest appreciation expressed in fees. There are men of high education and culture in the dental profession, who employ their education and engage their minds and best thought outside of their profession. Elihu Burritt, the learned blacksmith, employed his talent outside of his shop, and became master of fifty-two languages. But his linguistic learning added nothing to his work at the anvil. Hence his shop-work was not more appreciated because of his learning. The callings of some men of excellent minds and culture are such as to afford no scope for their study. They cannot put into their work the study and thought of which their minds are capable. They are compelled to go outside of their callings to find suitable employment for their minds. But this is not the case with dentistry and the dental profession.

When, in times past, dentistry was made simply a mechanical and manipulative art, there was little in it to invite the attention of men of good mental capacity who loved study. But since dentistry has grown into a deep and profound science, it affords scope for the best minds, and he who studies dentistry most is best capable of getting out of it what is most valuable and appreciable for mankind.

What is most valuable in dentistry is obtained only by study and research, and the value increases in proportion to the amount of brain-work employed in developing it. A shallow and superficial view of dentistry develops the shallow and superficial dentist, whose work is appreciated for all it is worth.

It is the comprehensive view of dentistry that inspires a man to enthusiasm in the pursuit of his profession, and to become the progressive man whom the intelligent and progressive people of the town like to meet and to employ. It is not enough, then, that a man should be educated and learned, but that he should be known as employing that education and learning enthusiastically in the pursuit of his profession, and not outside of it.

4. A dentist must have a high appreciation of *dentistry;* not a high appreciation of *himself* expressed in arrogant conceit, but a high appreciation of the value of dental science worked out in a dental practice for mankind. The man who knows most of dentistry must have the highest appreciation of its value to mankind.

It cannot be expected that the people, the laity outside the profession, will have a higher appreciation of dentistry than the dentist who represents the profession in the community. What they know of dentistry they learn from the dentists. Dentists are their only teachers. The truest value of the teeth can be learned only from dentists, for the reason that they have given the subject most attention.

The laity classify teeth as front and back teeth—esteeming that only the front teeth are of special value. The back teeth are regarded simply as a matter of convenience, or serving merely a mechanical support to the cheeks. If trouble occurs in a front tooth they are anxious to have it relieved, both to avoid the pain and a disfigurement of the face. But if a back tooth is in trouble and the pain of extraction is esteemed to be worse than the pain thus endured, extraction is decided upon without hesitancy. Then as to the molars as a whole—each one is regarded as of one-twelfth the value of the whole. So they are as ready to sacrifice a first molar as a third molar. How is a patron to learn that,—other conditions being equal—*a first molar is worth a hundred times as much as a third molar,* if some intelligent dentist does not explain it, and that in proportion to its value it is important to save it? How are they to understand that molars are worth a hundred times as much as incisors, unless some intelligent dentist shall take the trouble to explain the relations of the two classes of teeth to the economy of life and health?

If it can be shown to a patron that teeth have an intrinsic value—not a merely fictitious and fanciful value—such an one will be as ready to pay for securing such value as for obtaining any other value.

A lady singer came to my office with a troublesome lateral incisor, from which she had suffered greatly with an abscess at the root, and a worse trouble at the hands of a dentist who had drilled through the lateral wall of the canal and out through the alveolar process and the gum, at a point midway between the cervical margin and the apex of the tooth. I explained to the lady the nature of the trouble and the complications of disease involved, and she was disposed to have it out immediately. All her other teeth were good. I explained to her the effect of losing it—the change it might make in her voice—the trouble of a plate or of any other method of supplying an artificial tooth—that this trouble was not for a few days or weeks only, but for forty or fifty years, and as long as she lived—that there was no possible way of inserting a tooth without damaging other teeth—that she would mourn the loss of it all her days. She then asked what it would cost to treat it. With little thought of what it might cost, I replied, "It may cost you $20.00." Although it did cost, at ordinary charges, considerably more than that, as she was a girl without means except the earnings of her voice, I charged her no more than I at first intimated, which she pronounced very reasonable and which she did not hesitate to pay. She had on her lips the praise of the virtues of false teeth. Suppose I had silently accepted it? This case serves to show the importance of explaining, in the minutest particulars, the far-reaching damage incurred by the loss of the teeth. To escape this long train of evils, she was willing to pay as liberally as her means would allow.

How many people, outside the dental profession, know that there are diseased conditions in the mouth that lay the foundation for the worst forms of dyspepsia and a life-long trouble, and that this may begin in early childhood; that the nervous system may be completely undermined and the tortures of neuralgia be made a habitual complaint, from no other cause than diseased teeth; that nutrition may become so impaired by dental diseases that a skeletonized body may present itself daily at a bountiful table and remain skeletonized?

These well-known results of dental lesions must be made the theme of daily lectures at the dental chair. Every important operation should be of a clinical character, and made the basis of valuable instruction.

It is not expected that the lower classes will appreciate dentistry for more than the relief it gives to present suffering. Hence it is not uncommon to find persons who will not have a tooth treated and saved if it will cost more than to have it extracted. The paying patronage of dentists comes from the higher and more intelligent classes; not because of their wealth or their better learning, but their general intelligence and mental culture enables them to understand better, when explained to them, the true value of anything, and their better financial condition enables them to obtain what they most value. Upon this class it pays to bestow thought and time in giving intelligent instruction.

The point to be made is the value of dental operations in promoting personal comfort, health and longevity, the preservation of the form of the features, personal identity, and the pleasures of social intercourse without embarrassment, as well as presenting old age freed from its most unwelcome deformity.

The rule should be "line upon line, precept upon precept, here a little and there a little" of intelligent instruction touching the deeper and more important results of dental operations than those immediately experienced—results pertaining to the long future of their own lives and the well-being of their posterity, whose lives may be made miserable or pleasurable by inheritance.

A dentist's patrons must be made to feel that he is truly honest in his dental operations—that he is not working simply to get a living—that he does not talk dentistry for the mere dollar's sake, but that in his dental skill and knowledge he holds a high trust for the benefit of his fellow men. All kinds of trickery, deceit, and covering up of conditions or results, should be as foreign to him as to sun-light. All that he says and does should have the openness of christian day-light. This perfect frankness and honesty creates in the mind of a patron confidence and trust, which is the only foundation upon which can be built a successful practice.

